# Driving electrochemical reactions at the microscale using CMOS microelectrode arrays†

**DOI:** 10.1039/d3lc00630a

**Published:** 2023-10-31

**Authors:** Jens Duru, Arielle Rüfenacht, Josephine Löhle, Marcello Pozzi, Csaba Forró, Linus Ledermann, Aeneas Bernardi, Michael Matter, André Renia, Benjamin Simona, Christina M. Tringides, Stéphane Bernhard, Stephan J. Ihle, Julian Hengsteler, Benedikt Maurer, Xinyu Zhang, Nako Nakatsuka

**Affiliations:** a Laboratory of Biosensors and Bioelectronics, Institute for Biomedical Engineering, Eidgenössische Technische Hochschule (ETH) Zürich Switzerland nakatsuka@biomed.ee.ethz.ch; b Ectica Technologies AG Zürich Switzerland; c Macromolecular Engineering Laboratory, Department of Mechanical and Process Engineering, Eidgenössische Technische Hochschule (ETH) Zürich Switzerland

## Abstract

Precise control of pH values at electrode interfaces enables the systematic investigation of pH-dependent processes by electrochemical means. In this work, we employed high-density complementary metal-oxide-semiconductor (CMOS) microelectrode arrays (MEAs) as miniaturized systems to induce and confine electrochemical reactions in areas corresponding to the pitch of single electrodes (17.5 μm). First, we present a strategy for generating localized pH patterns on the surface of the CMOS MEA with unprecedented spatial resolution. Leveraging the versatile routing capabilities of the switch matrix beneath the CMOS MEA, we created arbitrary combinations of anodic and cathodic electrodes and hence pH patterns. Moreover, we utilized the system to produce polymeric surface patterns by additive and subtractive methods. For additive patterning, we controlled the *in situ* formation of polydopamine at the microelectrode surface through oxidation of free dopamine above a threshold pH > 8.5. For subtractive patterning, we removed cell-adhesive poly-l-lysine from the electrode surface and backfilled the voids with antifouling polymers. Such polymers were chosen to provide a proof-of-concept application of controlling neuronal growth *via* electrochemically-induced patterns on the CMOS MEA surface. Importantly, our platform is compatible with commercially available high-density MEAs and requires no custom equipment, rendering the findings generalizable and accessible.

## Abstract 2

Precise control of pH values at electrode interfaces enables the systematic investigation of pH-dependent processes by electrochemical means.

## Graphical abstract

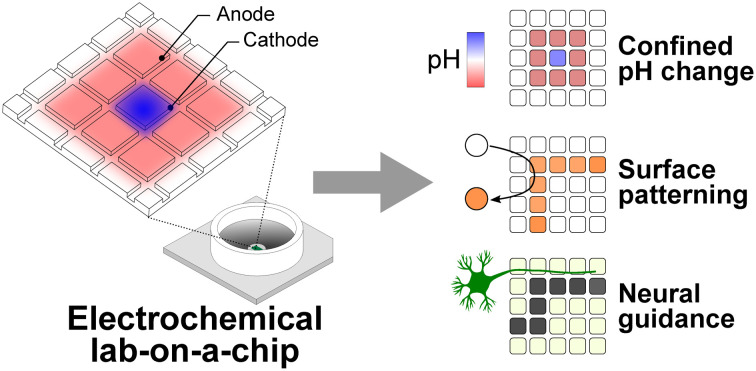

## 1 Introduction

In Nature, pH regulation plays a crucial role in various processes such as enzymatic activity^1^ and protein function.^2^ Controlled pH modulation enables studies on the effect of local pH changes on biological systems.^3^ A route to induce pH changes within a solution is by utilizing electrochemistry at electrode surfaces. Faradaic reactions occurring at the electrode–electrolyte interface alter the local hydrogen ion and hydroxide concentrations and in turn, the pH value in the electrode vicinity.^4–7^ Controlling the pH with high spatial resolution in a parallelized manner would allow the generation of multiple pH microenvironments on one chip. However, the confinement of induced pH changes in solution is challenging due to the diffusion of ions that are generated on the electrode surface.

While one approach to limit the spatial extent of electrochemically-induced pH elevation zones is to utilize buffers,^8,9^ this method hinders large pH changes in the buffered environment. To control the extent of pH microenvironments by solely electrochemistry, complementary metal-oxide-semiconductor (CMOS) technology has been used to manufacture a pH localizer-imager comprised of a 256-pixel array.^10^ Every pixel consists of an anode–cathode electrode pair for electrochemical pH control. By placing the anode and cathode directly next to each other, hydrogen ions generated at the anode can be captured at the neighboring cathode, leading to precisely defined regions of altered pH. This novel CMOS platform also utilizes electrochemical sensing techniques to monitor the induced pH changes. This system, however, is custom-made and limited to a spatial resolution of approximately 100 μm. In this work, we deployed commercially available CMOS microelectrode arrays (MEAs) with 26 400 electrodes to confine electrochemical reactions in a parallelized fashion at the smallest reported scale to date: the electrode pitch of 17.5 μm. As our approach requires no custom-built hardware, it is accessible to many groups using CMOS MEAs.

Such high-density CMOS MEAs are conventionally used as a tool to record extracellular signals from electrically active cells such as neurons *in vitro*.^11–13^ While keeping this application in mind, our work focused on harnessing the vast number and small electrode size to enable highly localized pH control at scales that push the current spatial limits. Selectively setting electrodes to cathodes and adjacent electrodes to anodes, we limit hydrogen and hydroxide ion diffusion in unbuffered environments. Further, the flexibility in electrode routings offered by the switch matrix beneath the CMOS MEA,^14^ enables modular patterning of practically any pH-driven image on the chip surface. We quantified the induced pH change optically using a pH-sensitive fluorescent reporter and generated a relationship between the applied voltage to the microelectrodes and the locally induced pH change.

On the surface of the CMOS MEAs, we demonstrate the capacity to conduct both additive and subtractive patterning at single-electrode resolution using pH control. Molecules can be coated on electrodes through electrodeposition where species in solution are oxidized and deposited onto the surface. An example of this chemistry is the direct oxidation of dopamine on the surface of a microelectrode to yield polydopamine,^15–17^ a mussel-inspired biopolymer that enables versatile surface coatings.^18,19^ Thus, for additive patterning, we electrodeposited polydopamine at specific microelectrodes on the CMOS MEA by generating pH values above the threshold for dopamine oxidation (pH > 8.5). For subtractive patterning on the CMOS MEA, we locally removed cell-adhesive polymers by applying an electric potential to selected microelectrodes.^20^ The resulting voids were then backfilled with antifouling molecules that have been shown to restrict neuronal overgrowth *in vitro*.^21^ We then influenced the growth of primary rat cortical neurons on electrochemically-patterned CMOS MEAs as a potential application of our system. Achieving distinct zones of altered pH values on CMOS MEAs could pave the way towards precisely defined cell guidance patterns created *in situ* and further establish a generalizable strategy to study pH-dependent processes on the microscale.

## 2 Materials and methods

### 2.1 Electrical setup

#### 2.1.1 CMOS microelectrode arrays

Commercially available CMOS MEAs (MaxOne, MaxWell Biosystems, Switzerland) were used in this work. The details of the system were previously described in the literature.^22^ Briefly, the CMOS MEA chip consists of 26 400 microelectrodes that are arranged in a 120 × 220 grid at a pitch of 17.5 μm. The microelectrode grid is surrounded by a circumferential reference electrode. Voltage signals from up to 1024 electrodes can be recorded in parallel. The CMOS MEA further contains three digital-to-analog converters (DAC) that can be programmed to provide independent electrical stimuli to microelectrodes. Multiple versions of the CMOS MEA were utilized in this work differing in electrode coating and surface topology. The chip's microelectrodes were either composed of bare platinum (Pt) or coated with platinum black (PtB), both of which were directly purchased from the supplier, MaxWell Biosystems. All data was generated using chips with PtB electrodes unless stated otherwise. The MEA surface topology was either non-planar as described in ref. 23 or flat as shown in ref. 24 based on availability from the distributor. For experiments conducted herein, the MEA surface topology did not affect the results. The flat MEAs (MaxOne+) feature electrodes of 10 × 10 μm^2^ in size in contrast to 9.3 × 5.45 μm^2^ on non-planar MEAs.

Scanning electron microscopy (SEM) images of the electrodes on non-planar chips are shown in the ESI† in Fig. S1. These images were taken on a Magellan 400 (Thermo Fisher Scientific) high resolution SEM. The beam voltage and current were set to 5 kV and 25 pA, respectively. The images were taken at a 40° and 0° tilt angle. The MEAs can be inserted into a recording unit, that is connected to a PC *via* a hub. Both the recording unit and hub were purchased from MaxWell Biosystems.

#### 2.1.2 Source measurement unit

A source measurement unit (SMU, Keithley 2636b, Keithley Instruments) was used for precise voltage control and current measurements. The SMU was connected to the recording unit *via* its external port and the voltage was set using a Python library.

#### 2.1.3 Electrochemical circuit

**Fig. 1:**
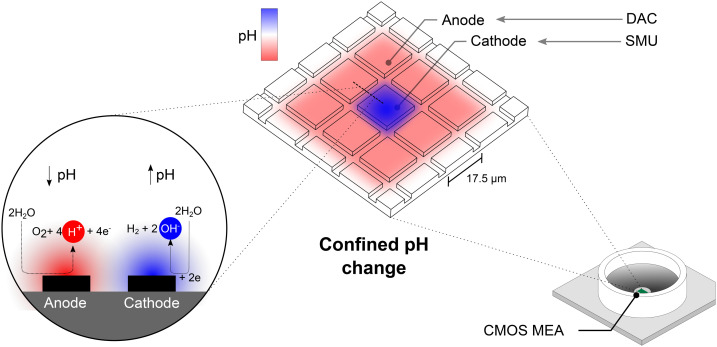
Inducing hydrolysis locally on CMOS MEAs yields spatially confined pH changes. The CMOS MEA enables simultaneous routing of practically any combination of electrodes. A voltage applied between two sets of electrodes forms an anode–cathode pair, at which hydrolysis can be induced. The electrochemical reactions yield the formation of hydroxide ions (OH^−^), which locally increase the pH. At the anode, the counter-reaction decreases the pH by generating hydrogen ions (H^+^). The potential on the cathode was set by an external SMU while the anodic potential was set by an internal DAC.

The recording unit provides ports that allow connection to external signal generators *via* a LEMO-BNC cable. Electrodes were routed using the Python API provided by MaxWell Biosystems. Cathodic electrodes were connected to an internal common node available on the chip. This common node was routed to the device's external port, which was connected to the SMU. The ground potentials of the SMU and the recording unit were connected through the LEMO-BNC cable. Anodic electrodes were either undefined and hence left floating or routed around the cathodic electrodes. If defined, the electrodes surrounding the cathodic electrodes were selected as well for routing to form a directly neighboring anode. To achieve this configuration, the amplifiers routed to the anodic electrodes were connected to a system-internal stimulation DAC. Three 10-bit stimulation DACs are available on the chip. When the DAC connected to the anodic electrodes is set to a value of 512, these electrodes are set to the mid-potential of the chip at 1.65 V. The mid-potential of 1.65 V is also referred to as the floating potential of the chip^25^ and results from the chip's internal power supply voltage of 3.3 V. This electrochemical setup resembles a two-electrode system, where the cathode acts as the working electrode and the anode as the counter electrode. No further reference electrode was placed in the bath. The equivalent electrical circuits are shown in the ESI† in Fig. S2. Using the SMU, the voltage between the anodic and cathodic sets of electrodes can be varied, which alters the extent of electrochemical reactions (*i.e.*, hydrolysis), which results in a local pH alteration. The concept of this confined pH alteration is visualized in Fig. 1. To determine the influence of applied voltage on the resulting pH on the chip, a voltage sweep was performed. For this, the voltage was gradually increased from 0–1.6 V in steps of 100 mV every 60 s. Here, the voltage is defined as the potential difference between the anode and the cathode.

### 2.2 Solutions

#### 2.2.1 Buffer solution

All results in this work were obtained using a buffer solution unless stated otherwise. The usage of a buffer counteracts the induced pH changes but allows the manual adjustment of the starting pH of any precursor solution. A mixture of two buffers was utilized in this work. Sodium acetate (S7899) and HEPES (83264, both purchased from Sigma-Aldrich) were mixed and added to deionized ultra-pure water (18.2 MΩ cm^−1^ Milli-Q, Merck-MilliPore) to yield a final concentration of 0.125 μM for each component. The low buffer concentration was chosen to minimize any counteracting effects on electrochemically-induced pH changes, while still allowing manual adjustment of the initial pH of the bath solution. The buffering zone of sodium acetate ranges from pH 3.8 to 5.6 and provides a lower-bound barrier to avoid the potential denaturation of enzymes. HEPES provides a useful pH range of 6.8–8.2 and avoids overshooting and more precise pH control. Using an electronic pH meter (SevenEasy pH meter, Mettler-Toledo, Switzerland and MI-410 pH probe, Microelectrodes Inc., USA), the pH value of the buffer-containing solutions was manually adjusted with 0.1 M sodium hydroxide and 0.1 M hydrogen chloride.

#### 2.2.2 pH-sensitive dyes

**Fig. 2:**
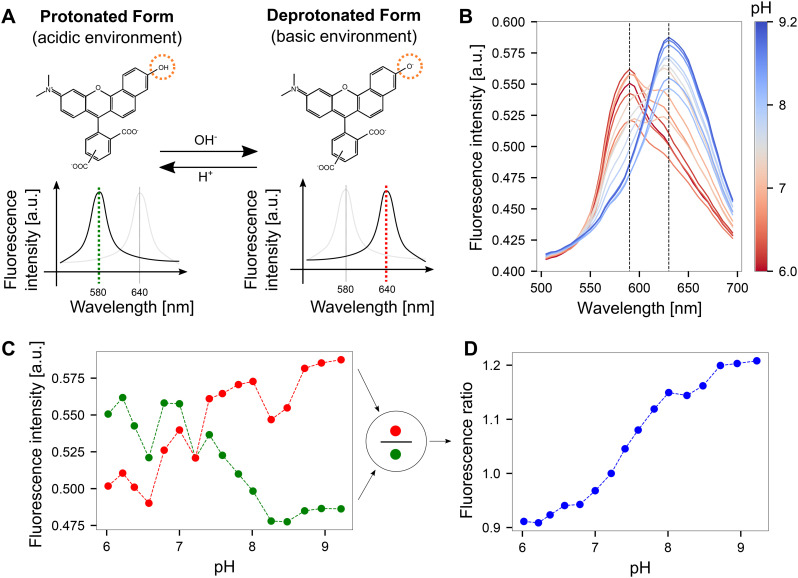
Quantifying pH optically on CMOS MEA surfaces using SNARF. A The fluorescence emission spectrum of SNARF is dependent on the pH of the environment. In an acidic environment, the emission maximum lies at 580 nm. This peak shifts to 640 nm in a basic environment. The structural change of the molecule was adapted from ref. 30. B Fluorescence emission spectra obtained by imaging SNARF solutions with known pH values on the CMOS MEA. The dashed lines indicate the local maxima of the SNARF emission spectra. C Fluorescence intensity obtained from the emission spectra at the dashed lines shown in B for both the red and green windows. D Dividing the fluorescence intensities from the red *vs.* green windows yields fluorescence ratios that can be mapped to corresponding pH values.

**Fig. 3:**
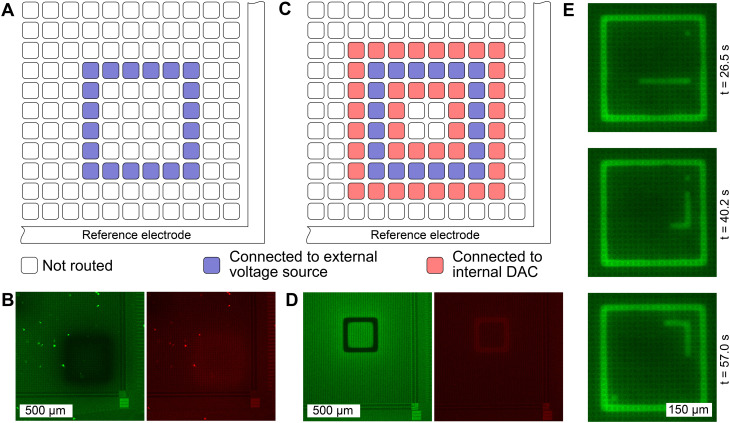
The spatial extent of the induced pH change can be confined precisely. A Schematic of a routing in which electrodes are connected to an external voltage source, while all other electrodes are left floating. B Resulting fluorescence images with SNARF as the fluorescent reporter using the routing shown in A with an applied voltage of 1.6 V. The induced pH change is diffuse, lacking spatial confinement. The green image shows the fluorescence intensity within a 10 nm window centered around the 580 nm emission peak while the red image shows the fluorescence intensity around the 640 nm peak. C Schematic of a routing where neighboring electrodes are connected to a low-impedance sink, *i.e.*, to a DAC that is set to the mid potential of the chip at 1.65 V. D Applying a voltage of 1.6 V between the two electrode combinations, yields a precisely confined pH change. The reduced fluorescence intensity in the green window and the increase in the red window, demonstrate the fluorescence emission shift of SNARF that occurs when the solution shifts toward a basic environment. E Switching between predefined electrode configurations allows changing the locations of pH induction quickly in sequence. Here, three frames of a simulated game of *Snake* utilizing FITC as the fluorescent pH dye are shown. The “snake”, “food”, and square boundary comprise the cathode, and all other electrodes form the anode.

We used carboxy-seminaphtharhodafluor (5-(and-6)-carboxy SNARF-1, C1270, Thermo Fisher), a fluorescent probe used for intracellular pH analysis^26,27^ to quantify induced pH changes on the MEA surface similarly to Frasconi *et al.*^3^ The local pH was determined optically using a ratiometric method to quantify the fluorescent emission of SNARF. In an acidic environment, SNARF is present in a protonated form that yields a fluorescent emission spectrum with a peak at 580 nm when excited with a laser of 488 nm wavelength. In basic environments, SNARF switches to a deprotonated form with a shift in the emission spectrum peak to 640 nm. This pH-dependent shift in the emission spectrum of SNARF is shown in Fig. 2A. Upon dividing the fluorescent intensity values detected at these two peak wavelengths, a fluorescent intensity ratio as a function of pH was obtained. The SNARF was dissolved in deionized ultra-pure water and stored at −20 °C in aliquots at a concentration of 220 μM. The SNARF aliquot was thawed at room temperature and diluted with the buffer solution to yield a final working concentration of 55 μM prior to use. To calibrate each CMOS system, *i.e.* obtaining a relationship between the local pH and the fluorescent ratio, the SNARF solution was manually adjusted to the desired pH within a window of pH 6–9 in 17 steps (full calibration) or 3 steps (3-point calibration) and then pipetted onto PtB chips for subsequent imaging. For the analysis of the temporal behavior when pH patterns are quickly altered on the CMOS MEA (Fig. 3E), fluorescein-5(6)-isothiocyanate (FITC, F3651, Sigma-Aldrich) was used at a working concentration of 20 μM. While less precise than SNARF, FITC was chosen for enhanced visualization as its fluorescence emission intensifies with increasing solution pH within the window of interest.^28^

#### 2.2.3 Dopamine solution

To form polydopamine locally on the MEA surface, a precursor dopamine solution was prepared consisting of 2 mg mL^−1^ dopamine hydrochloride (H8502, Sigma-Aldrich) in the aforementioned buffer solution. The solution was adjusted to a pH of 5.8 using HCl and NaOH to avoid the spontaneous formation of polydopamine, which forms most efficiently at a pH above 8.5.^19^

### 2.3 Surface patterning on the MEA

#### 2.3.1 Initial CMOS MEA surface treatment and PLL removal

The CMOS MEAs were coated with FITC-tagged poly-l-lysine (PLL-FITC, P3069, Sigma-Aldrich), which is widely used as a surface coating to promote neural adhesion. A 50 μL drop of 0.1 mg mL^−1^ PLL-FITC was pipetted onto the center of the CMOS MEA to cover all electrodes and surrounding areas. After 30 min the droplet was aspirated, the surface was rinsed three times with ultrapure water, and blow-dried with N_2_. For the local removal of PLL-FITC, the CMOS MEA was inserted into the recording unit and connected to the SMU as described before. A 23 × 23 electrode patch was routed, out of which an 11 × 11 electrode in the center was defined as a cathode, leaving 408 electrodes as an anode. A potential of 10 mV was defined on the SMU, and the DAC was set to the mid-potential of the chip at 1.65 V resulting in an applied voltage of 1.64 V. This voltage was applied for 60 min. After 30 min the water was exchanged with fresh water to remove PLL-FITC monomers that were removed from the surface. After another 30 min the chip surface was rinsed three times with water.

#### 2.3.2 Backfilling with antifouling coatings

The antifouling polymer, poly(acryl-amide)-*g*-(PMOXA100, 1,6-hexanediamine, 3-aminopropyldimethylethoxysilane, rhodamine B), called PMOXA for brevity, was used to backfill regions of removed PLL. PMOXA is known to prevent neural adhesion to surfaces.^21^ The polymer is labeled with a rhodamine tag to render it fluorescent. PMOXA was used at a concentration of 0.1 mg mL^−1^ in deionized water and incubated on top of the dry MEA surface for 30 min at room temperature. Then, the solution was aspirated and the CMOS MEA surface was rinsed three times with ultrapure water before a final blow-drying step with N_2_.

#### 2.3.3 Polydopamine deposition

A 50–100 μL drop of the dopamine solution was pipetted onto a FITC-PLL coated MEA before a voltage between 1.1–1.5 V was applied to the system for 20–30 min. Then, the non-crosslinked dopamine was aspirated from the CMOS MEA surface and rinsed three times with ultrapure water before the surface was blow-dried with N_2_.

#### 2.3.4 Cell seeding

To visualize the impact of PLL removal and PMOXA addition on the growth of neurons on the MEA surface, primary rat cortical neurons obtained from E18 Sprague-Dawley rat embryos (Janvier Labs, France) were used. Cells were dissociated according to a previously published protocol.^29^ The veterinary office of the canton Zurich reviewed and approved the use of animal cells. The seeding density ranged from 10 000–30 000 cells per CMOS MEA which translates to 50 000–150 000 cells per cm^2^.

### 2.4 Imaging techniques

#### 2.4.1 Confocal laser scanning microscopy and optical pH detection

The fluorophore-based pH measurements were conducted using a confocal laser scanning microscope (CLSM, FluoView 3000, Olympus). The opaque CMOS MEA was flipped to be accessible by the inverted microscope for imaging. The method was explained in detail in a prior publication.^23^ Briefly, a solution (either containing pH indicator or culture medium) was pipetted onto the chip and then a glass coverslip (12 or 14 mm diameter) was placed on top. Upon aspirating the excess solution around the coverslip, the coverslip was lowered until contact was made with the conic epoxy surrounding the CMOS chip. This approach allows imaging the chip surface with the 10× objective of the microscope while maintaining a small volume of liquid on top of the MEA surface. A laser with a 488 nm wavelength was used to measure the pH on the MEA surface using SNARF. Lambda scans were performed to obtain a coarse emission spectrum. For this, images were obtained in a window ranging from 500–700 nm in bins of 5 nm, resulting in 39 individual images. As this process is time intensive and the spectra are only evaluated at the wavelengths corresponding to the emission maxima of SNARF, double-window imaging was also conducted, where only two images were obtained. The fluorescent signal was recorded in a 635–645 nm window (named “red window” in this manuscript) and in a 575–585 nm window (“green window”). The fluorescent intensities within these channels were then used to determine the pH using a ratiometric method. When a voltage sweep was performed to determine the influence of applied voltage on the pH, images were continuously acquired every 10–15 s. To study the temporal behavior when switching between electrode configurations and hence pH patterns, the resonant scanner of the CLSM was used allowing the acquisition of images with a frame rate of 15 Hz. Surface patterns of PLL and PMOXA were imaged using two lasers and two detection windows in sequential imaging mode. The FITC-labeled PLL was exited with a 488 nm laser and the fluorescence emission was detected in a 500–540 nm detection range (named “FITC detection range” herein). The rhodamine-labeled PMOXA was exited using a 561 nm laser while detecting the fluorescence in a 570–670 nm detection range (named “PMOXA detection range” herein).

#### 2.4.2 Neuron imaging

To visualize the growth of neurons on the CMOS MEA, the neurons were stained fluorescently after at least 7 days *in vitro* (DIV). Alive cells were either stained using calcein-AM (C1430) or CellTracker Green CMFDA Dye (C7025) and dead cells were visualized using ethidium homodimer (L3224, all chemicals purchased from Thermo Fisher). The dyes were pipetted into the culture medium on top of the CMOS MEA to yield a working concentration of 1 μM. After an incubation time of 30 min, the culture medium was replaced with fresh medium.

## 3 Results and discussion

### 3.1 SNARF as a quantitative pH indicator on CMOS MEAs

We determined the induced pH changes in the vicinity of the microelectrodes on the CMOS MEA using SNARF, a pH-sensitive fluorescent indicator. To relate the fluorescent emission from SNARF to a quantitative pH value, a calibration step was first performed. Results from lambda scans at each pH step demonstrated that the emission spectrum of SNARF shifts with increasing pH (Fig. 2B). Extraction of the fluorescent intensity values at the maxima of the spectra at 580 nm and 640 nm (dashed lines in Fig. 2B) yielded the plot shown in Fig. 2C. An increase in fluorescence was observed in the red window (635–645 nm) as opposed to a reduction in fluorescence in the green window (575–585 nm). The few unexpected jumps in the curves are likely due to slight changes in the focal plane induced during liquid exchange and re-mounting the sample. By dividing the fluorescence intensity in the red window by the green, a fluorescence ratio was calculated and a continuous curve was obtained (Fig. 2D). This pH-dependent curve exhibits a key advantage of the ratiometric method: the ability to cancel out drifts of the focal plane. A more linear relationship between pH and fluorescence ratio was observed when the images were acquired using the dual-window approach rather than the lambda scan (Fig. S3†).

Since the full calibration process was time-consuming due to the use of 17 different solutions differing in pH and a linear trend was evident, we switched to the three-point calibration method where the fluorescence emission was recorded at pH values of 6, 7.5, and 9 on two CMOS MEAs at three different locations on the surface. The data points were fitted linearly as shown in Fig. S4.† This resulting calibration curve can then be used to translate a fluorescent ratio from SNARF into a pH value. Obtaining a precise relationship between pH and fluorescent emission proved challenging due to chip-to-chip surface variations. Control cyclic voltammetry measurements were performed to ensure that SNARF is not directly reduced or oxidized at the electrode surface and hence interfering with the optical pH determination (Fig. S5†).

### 3.2 Precise confinement of induced pH change at CMOS MEAs

To evaluate the diffusion characteristics of the induced pH change on the MEA surface, the electrodes were configured to induce local pH changes with SNARF solution at a starting pH of 5.8. While a pH change was observable when only the cathode was defined and connected to an external voltage source (Fig. 3A), spatial confinement was not achieved (Fig. 3B). We observe in this case, that the circumferential reference electrode is serving as the anode. However, as this reference electrode surrounds the exterior of the microelectrode grid,^22^ the large distance between the cathode (microelectrodes) and the anode (circumferential reference electrode) leads to unconfined diffusion, hindering precise control in pH patterning. When the anode was defined by connecting neighboring electrodes to an internal DAC, which was set to the mid-potential of the chip (Fig. 3C), a precise zone with elevated pH was established (Fig. 3D). Here, a square with a width of 3 electrodes (= 52.5 μm) was defined as a cathode (region of desired pH elevation) and a voltage of 1.6 V was applied between the anode and the cathode. The characteristic change in fluorescence emission of SNARF is evident. Upon increasing the pH, the fluorescence emission was reduced in the green window, while an increase was observed in the red window. A video illustrating the change in fluorescence of SNARF on the CMOS MEA while sweeping the input voltage is provided in the ESI† (SM1). Hydrolysis-induced bubble formation was not observed.

Moreover, we demonstrate the possibility of generating pH patterns in sequence in real time. We have simulated a game of “snake” in which 300 electrode configurations were generated and stored on the recording PC. Each configuration contained information on which electrode was defined as a cathode and connected to the SMU (snake, food, and game boundary), and which electrode was connected to the internal DAC to be set as an anodic “background”. The change in pH was visualized using an alternative fluorophore, FITC. While FITC does not allow quantitative pH determination due to single wavelength emission, visualization is enhanced as fluorescence emission increases linearly with pH. Each configuration was loaded onto the CMOS MEA for approximately two seconds. Three frames of the gameplay are shown in Fig. 3E and the full video can be found in the ESI† (SM2).

### 3.3 Electrochemical induction of an expansive pH range

**Fig. 4:**
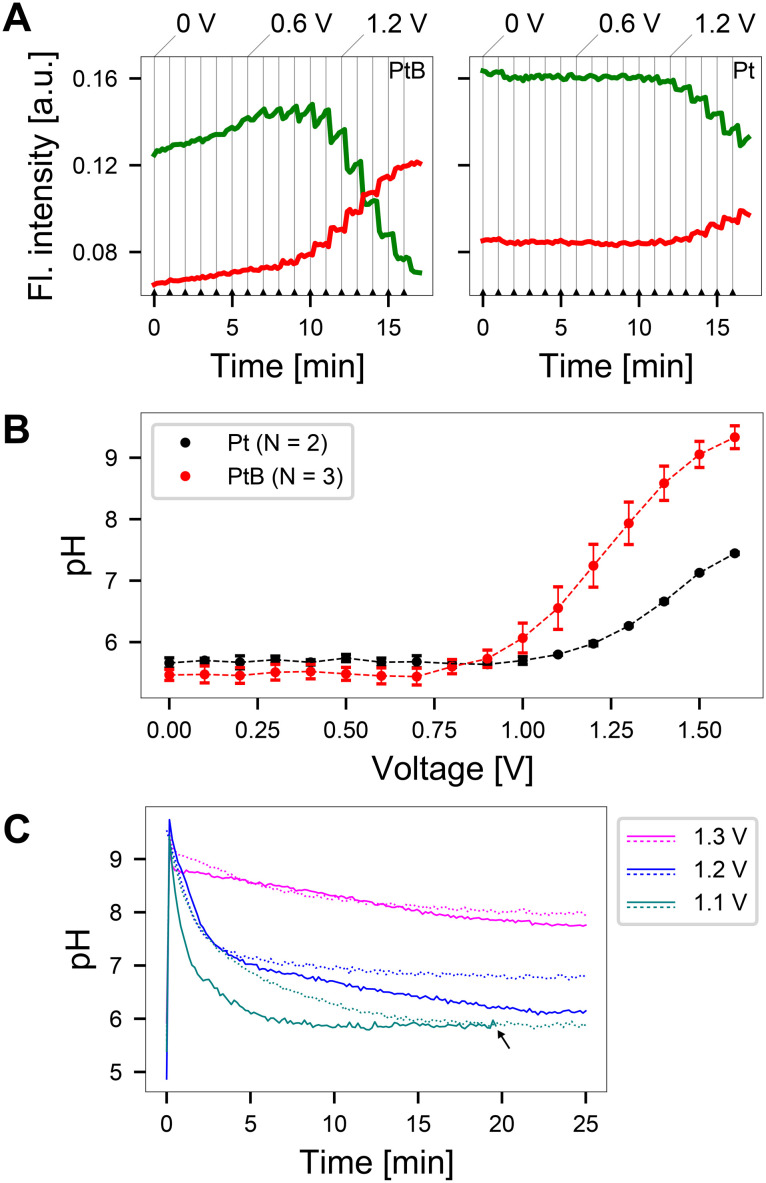
Relationship between applied voltage and induced pH changes on platinum black (PtB) and platinum (Pt) coated microelectrodes. A Increasing the applied voltage in steps of 0.1 V after 1 min yielded large changes in the fluorescence emission within the green and red emission windows when PtB electrodes were used. In comparison, Pt electrodes induced smaller changes. The grey vertical lines indicate time stamps at which the applied voltage was increased. B The fluorescence emission ratio from the data as shown in A yields a curve that relates the applied potential to the induced pH. A large pH range (6–9) can be covered using PtB electrodes, while Pt electrodes are limited in pH range with a maximum pH of around 7 when the highest voltage was applied. Shown here are the mean values and the standard deviation for *N* = 2 Pt and *N* = 3 PtB chips. C Temporal behavior of the induced pH change after the application of an external voltage for two PtB CMOS MEAs. The solid and dotted lines indicate data from two different chips. The initial pH was set to 5.8 and a voltage between 1.1–1.3 V was applied. The induced pH changes at 1.3 V showed decay to pH 8 within a 20 min window, while at 1.1 V the induced pH change decayed to a value around pH 6, close to the initial solution pH value. At 1.2 V, a decay to initial pH was observed on one chip (continuous blue line), while a steady pH around 7 was achieved on another chip (dashed blue line). The arrow indicates the formation of an air bubble under the microscope, which yielded the termination of the experiment after 20 min.

Using the electrode configuration shown in Fig. 3C, precise zones of pH elevation were generated. By selecting regions of interest within these zones in the acquired images, the fluorescence ratio was obtained and translated into an induced pH value. To relate the induced pH to the applied voltage, a voltage sweep was performed from 0 to 1.6 V upon incubating SNARF solution at pH 5.8 on the CMOS MEA. The fluorescence intensity values within the regions of interest (cathode), are shown in Fig. 4A and S6.† The vertical gray lines indicate the time stamps at which the voltage was increased by 0.1 V. The typical SNARF behavior was observed: a reduced fluorescence intensity in the green window accompanied by a rise in the red window with increasing pH. This behavior was especially pronounced on platinum black (PtB) chips compared to platinum (Pt) chips, where only a gradual change in fluorescence is observed. The variation in pH sensitivity is likely attributed to the increased electrode surface area resulting from higher porosity and roughness in PtB-coated electrodes^31^ when compared to pure Pt electrodes (as depicted in Fig. S1†). This alteration in the diffusion field and detection volume affects the mass transport of ions to the electrode surface.^32^

Evaluating these fluorescence emissions 30 s after the voltage was applied and using the calibration curve to convert the fluorescence ratio to pH, yields the result shown in Fig. 4B. We observed a large window of possible pH values on PtB chips that surpass a pH of 9, after which SNARF is no longer sensitive. In contrast, Pt chips reach a maximum value of pH 7.3 at the highest applied voltage. Furthermore, the change in pH observed at lower potentials in PtB electrodes compared to Pt surfaces, can be attributed to their enhanced catalytic properties, which accelerate electron transfer, thereby reducing the overpotential required for electrochemical reactions.^33^ The increased catalytic activity on the surface of PtB electrodes increases water oxidation, leading to a local pH increase at lower potentials. Voltages below 1 V showed negligible long-term pH change.

To analyze the stability of the induced pH change over time, voltages ranging from 1.1–1.3 V were applied to two PtB MEAs. This range was chosen for real-time measurements as voltages below 1 V induced minimal changes in the pH values (Fig. 4A). We observed an initial overshoot and subsequent decay in the fluorescence emission at all voltages tested (Fig. 4C). Such overshoots were also observed at voltages as low as 0.3 V (small spikes in the green and red window fluorescence in Fig. 4A). This phenomenon likely arises from oxygen reduction, which consumes hydrogen ions, that occurs when voltages are first applied at microelectrodes.^34^ This hydrogen ion depletion leads to a sudden spike in pH, which gradually decays to reach equilibrium. Upon application of 1.1 V, an overshoot to a pH > 9 is followed by subsequent decay to pH > 6, the starting pH of the solution. Negligible changes in pH were observed upon applying a voltage of 1.2 V, which is slightly below the water-splitting window, on one MEA (continuous blue line). Another chip stabilized at a pH value of 7 upon the application of 1.2 V (dashed blue line). In contrast, when 1.3 V was applied to the system, equilibrium was reached at pH 8. These measurements indicate that reproducible pH values can be achieved when voltages outside the water window are applied. The induced pH changes were dependent on the starting pH of the solution as well as the buffer concentration (Fig. S7†).

### 3.4 Performing additive and subtractive patterning *in situ*

**Fig. 5:**
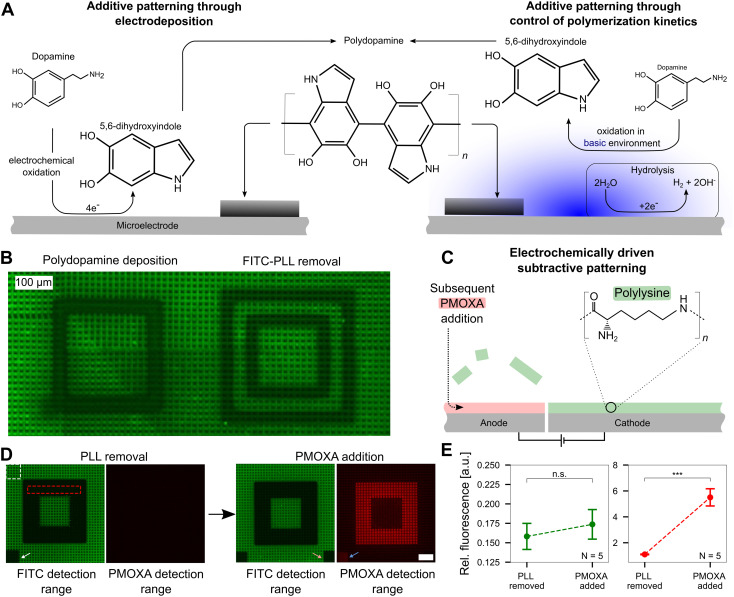
Precise pH control allows additive and subtractive patterning at the single-electrode level. A Additive patterning using polydopamine. Left: Forming polydopamine through direct oxidation of dopamine. At the electrode, dopamine is electrochemically oxidized forming 5,6-dihydroxyindole, which then crosslinks to form polydopamine that deposits on the electrode surface (black coating). Right: Polydopamine formation through oxidation of dopamine in a basic environment. Inducing hydrolysis yields an increased pH at the cathode, which subsequently yields the oxidation of dopamine to 5,6-dihydroxyindole and the formation and deposition of polydopamine. The figure is adapted from previous literature ref. 16 and 36. B Additive and subtractive patterning on a CMOS MEA that was previously coated with fluorescein (FITC)-labeled poly-l-lysine (PLL). Left: Deposition of polydopamine on the cathode. Right: Delamination of PLL on the anode. C Electrochemically driven subtractive patterning. Applying a large enough positive electric potential at a microelectrode leads to the delamination of previously bound FITC-PLL. The void can subsequently be filled with another molecule, such as an antifouling polymer, PMOXA. D Removal of FITC-PLL and replacement of PMOXA. The PMOXA is rendered fluorescent using rhodamine and hence yields a fluorescence signal on the CMOS MEA. The white (reference) and red (signal) dashed boxes indicate the regions of interest for the analysis shown in E. E Quantitative analysis of the PMOXA addition. The relative fluorescence intensity within the FITC detection range did not change significantly (*t*-test, *p* > 0.2) indicating the PLL replacement outside the routed electrodes is negligible. The increase in fluorescence in the PMOXA detection range demonstrates PMOXA adherence to the anodic electrodes (*t*-test, *p* < 0.001).

Upon understanding and quantifying the extent of pH control in a voltage-dependent manner on the surface of CMOS MEAs, we investigated the ability to harness this electrochemical setup for surface patterning. For additive patterning, we exploited the pH-dependent oxidation process of dopamine to polydopamine, where polymerization is initiated when a pH threshold of ∼8.5 is surpassed (Fig. 5A).

While the exact mechanism of polydopamine formation is still unknown,^35^ based on prior reports, we hypothesize that one of two mechanisms (or a combination thereof) are happening at microelectrode surfaces. First, dopamine can be electrochemically oxidized on an electrode to 5,6-dihydroxyindole, which can further crosslink to form polydopamine, an adhesive polymer that deposits on the MEA.^16^ Second, dopamine can be pre-dissolved in a basic environment and undergo oxidation over time, eventually yielding the formation and deposition of polydopamine.^36^ Both pathways are shown in Fig. 5A in a simplified form. As dopamine polymerization is known to be buffer dependent,^37^ we first confirmed that this process can occur in our specific buffer, a mixture of HEPES and sodium acetate. In a buffer solution adjusted to pH 8.5, monomeric dopamine was incubated. The solution turns black within a few hours (not shown), confirming the generation of polydopamine. As polydopamine is highly light absorbing, the color transition of the dopamine solution can be used as an indicator of its formation.^38^

Upon confirming that polydopamine can form in the buffer solution used in our experiments, we investigated additive polydopamine patterning at specific microelectrodes on the chip *in situ*. Prior to polydopamine polymerization, the chip was coated with FITC-PLL to visualize the polydopamine, which is highly light absorbent^39^ and a fluorescence quencher.^40^ Thus, polydopamine formation will quench the fluorescence of the FITC-PLL on the surface to reveal patterned locations. Dopamine solution (pH = 5.8) was incubated on the chip surface and a voltage of 1.4–1.5 V was applied in a square pattern to induce pH values >8.5 at localized positions. A black square is observed as a result of the polydopamine patterning (Fig. 5B, left side). An image of the whole CMOS MEA surface with multiple polydopamine patterning sites is shown in the ESI† (Fig. S8). As a control, only the buffer without dopamine present was incubated on the chip surface with the same protocol of applied voltage. In contrast to the polydopamine square, a hollow square appears, indicating that the PLL was removed from the chip surface (Fig. 5B, right side) at anodic electrodes. This PLL removal effect is not observed when dopamine is present in solution, which may be due to the direct electrochemical oxidation of dopamine on anodic electrodes (left pathway, Fig. 5A), which blocks PLL removal.

This effect of removing polymers adhered to the MEA surface by electrochemical means, suggested that our platform is suitable for subtractive patterning. Upon applying an electrical potential (1.64 V for 60 min) between two sets of electrodes, positively charged FITC-PLL can be removed from the anode (positive electrode) through electrostatic repulsion (Fig. 5C and Video SM3 in the ESI†). After the removal of the FITC-PLL, the voids can be backfilled with another polymer. Herein, we replaced the FITC-PLL with the antifouling polymer, PMOXA, which was labeled with rhodamine (that has an emission peak at a different wavelength in contrast to FITC at ∼580 nm) for optical detection. This surface pattern was chosen as a candidate for neural patterning: PLL promotes neuron growth while PMOXA prevents cell adhesion. Fig. 5D shows the patterning approach with subsequent PMOXA addition. After removal of PLL in a 6-electrode wide (105 μm) pattern (electrode schematics shown in Fig. S9†), a dark square appears, confirming that the FITC-PLL was removed. A negligible change in fluorescence is visible in the PMOXA detection range. However, upon backfilling with rhodamine-linked PMOXA, fluorescence in the same area appears in the red PMOXA detection range.

The white arrow in Fig. 5D points to a small square on the MEA surface that was photobleached by shining the laser for 2 min prior to PMOXA addition. The orange arrow indicates a bleached region post PMOXA incubation. Bleaching the chip prior to PMOXA addition leads to visible fluorescence in the PMOXA detection range (blue arrow) after PMOXA incubation. A possible explanation of the increase in fluorescence in the photobleached area may be the partial removal of FITC-PLL from the microelectrode driven by reactive species that are generated during the photobleaching process.^41^ Evaluating the fluorescence intensities in all images in the red dotted square and normalizing with intensities in the area outside the altered fluorescence (white dotted area) yields the data shown in Fig. 5E. No significant difference in the fluorescence emission within the FITC detection range is observable (*p* > 0.2, *N* = 5 chips) indicating that there is negligible displacement of FITC-PLL with PMOXA. In contrast, the increase in fluorescence within the PMOXA detection range is significant (*p* < 0.001, *N* = 5 chips), confirming that PMOXA was immobilized in the areas where FITC-PLL was previously removed. The fluorescence images for all five CMOS MEAs are shown in Fig. S10.†

### 3.5 Surface patterns influence the growth of neurons

**Fig. 6:**
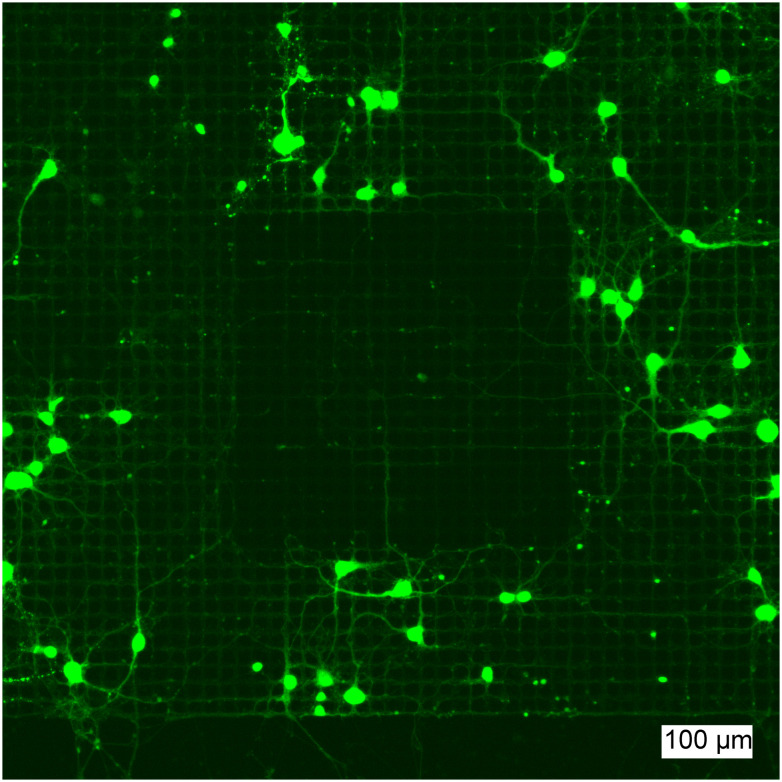
Neurons following the induced surface pattern on top of the CMOS MEA surface. Cortical neurons were seeded onto a CMOS chip on whose surface PLL was locally removed in the shape of a square. The resulting void was backfilled with PMOXA. The cortical primary rat neurons were rendered green fluorescent using CMFDA and imaged at DIV 14. No soma are visible within the square while axons were able to grow inside the square in between electrodes.

Neurons were grown on the CMOS MEAs after FITC-PLL and PMOXA were localized in specific regions to interrogate the potential of these surface patterns to influence neuronal propagation. When neurons were seeded onto these PLL/PMOXA square patterns, the neurons preferentially adhered outside the PMOXA-coated region (Fig. 6). This observation confirms the potential of this patterning strategy to harness the properties of different polymers: PLL promotes while PMOXA impedes the adhesion of neurons and neurite growth. Interestingly, we observed that single neurites can grow on the patterned regions in between the microelectrodes. This effect likely arises due to PLL remaining in the areas outside of the voltage-applied microelectrodes where PLL is replaced by PMOXA. To this point, the surface topology of the chip itself can provide axonal guidance. We have observed that this effect is highly dependent on the cell density where high neuron counts lead to clustering of neurons that can bridge surface patterns more easily (Fig. S11†). Thus, control of neuron seeding densities is critical for electrochemically-generated polymer patterns to influence neuronal growth.

## 4 Conclusions and outlook

In this work, we demonstrated the potential of CMOS MEAs to serve as modular platforms that can control electrochemical reactions precisely with unprecedented spatial resolution and flexibility. We were able to drive electrochemical reactions *in situ* using various combinations of microelectrodes using commercially available CMOS MEAs intended for electrophysiology with 26 400 electrodes at 17.5 μm pitch. We introduced a method to induce locally confined pH changes on the CMOS MEA by defining anode–cathode pairs in direct proximity. Regions of elevated pH on the anode are confined by capturing diffusing hydroxide ions at the neighboring cathode. Through this approach, we derived the relationship between applied electrical voltage and induced pH change on CMOS MEAs.

Compared to recently published platforms for local pH control,^10^ our concept offers an order of magnitude improvement in spatial resolution by confining electrochemical reactions at the scale of single electrodes. However, our approach does not provide a direct electrochemical pH measurement on site, which necessitated the use of a fluorescent pH reporter, SNARF, to quantify induced pH changes at microelectrode surfaces. In exchange, our method is compatible with existing hardware of CMOS MEA systems; the spatial resolution is solely limited by what is currently available on the market. A recent report introduced CMOS MEAs with 236 880 electrodes distanced only 250 nm from each other.^42^ Such scaling of electrode size and number holds promise to push the limits of the spatial resolution of our presented methods.

Moreover, we showed the capacity to assemble polymers at localized regions on the surface of CMOS MEAs through additive and subtractive patterning techniques. Additive patterning was demonstrated through the local formation of polydopamine on selected microelectrodes. While polydopamine formation through electrodeposition has been reported in the literature on the surface of passive low-density MEAs,^15,16^ our approach demonstrated a significantly improved control of polydopamine deposition at the resolution of individual microelectrodes. In addition, as polydopamine formation is time and pH dependent, our platform could be used to control the thickness of polydopamine films deposited on microelectrodes, which may alter the adhesive properties of the film. Furthermore, subtractive patterning was achieved through the removal of PLL, a polymer coating often used to render surfaces adhesive to neurons. While electrochemical removal of PLL from surfaces has been explored,^20^ our strategy enables PLL removal with improved precision.

By backfilling the voids with a cell-repellant polymer, PMOXA upon precise removal of PLL, we investigated the potential of this generated pattern to influence the growth of primary rat neurons. We observed that neurons avoided regions patterned with PMOXA and preferentially grew on locations with PLL. While this patterning approach on MEAs is less effective than the use of microstructures,^23,24,43,44^ our results serve as a proof-of-concept that surface patterns on MEAs can be induced *in situ* to introduce neural guidance at unprecedented spatial resolutions *in vitro*. Microstructures excel in the guidance of neurons due to their three-dimensional physical confinement of neurons.^45,46^ The growth of such structures in real time on the CMOS MEA would enable highly modular neural guidance.

Hydrogels are promising candidates to form such structures on the CMOS MEA using our method: the enzymatic crosslinking of hydrogels is a pH-dependent process that is most efficient around neutral pH.^47^ We conducted preliminary attempts at controlling the kinetics of hydrogel formation by local pH modulation. Upon seeding neurons on the surface post-hydrogel formation, evident obstruction of neuronal growth was observed based on the extent of polymerization time (Fig. S12†). However, this process was not reproducible enough, and further investigations are necessary to optimize the system for neuronal patterning, which was beyond the scope of this work. Our primary objective was to develop and validate a highly flexible lab-on-a-chip system to drive electrochemical reactions at the microscale, using a scalable method that has the potential to pattern batches of chips.

Further, our approach may provide a platform to influence cells already present on the chip surface. For example, driving a continuous direct current between two electrodes has been shown to induce lesions in nerves, disrupting motor activity signaling.^48^ Interacting with nerve cells on CMOS MEAs may lead to improved understanding of therapeutic strategies to treat pain. Moreover, as electrochemical reactions can lead to complete oxygen depletion at the surface of addressed electrodes, the presented approach could be used to induce local hypoxia.^34^ Studies have suggested that hypoxia may preserve or enhance stem cell phenotypes for both normal and cancerous cells^49^ – a phenomenon that could be interrogated on chip. Our use of commercially available hardware enables translation of our findings to a wide range of research laboratories.

## Author contributions

JD: conceptualization, data curation, formal analysis, investigation, methodology, software, supervision, visualization, writing – original draft. ARu, JL, MP, LL, AB, MM, ARe: data curation, formal analysis, software, validation, visualization. CF: conceptualization, data curation, investigation, methodology, software, resources, supervision, validation. BS: conceptualization, investigation, methodology. CMT, SJI: investigation, methodology. SB, JH, BM, XZ: investigation. NN: conceptualization, investigation, methodology, project administration, supervision, validation, writing – original draft.

## Conflicts of interest

The authors have no relevant financial or non-financial interests to disclose.

## Supplementary Material

LC-023-D3LC00630A-s001

LC-023-D3LC00630A-s002
