# Supplementary material for: Driving electrochemical reactions at the microscale using CMOS microelectrode arrays
Source: Lab Chip. 2023 Oct 31;23(23):5047–58. doi: 10.1039/d3lc00630a (PMC10661664; doi:10.1039/d3lc00630a)
Supplement: LC-023-D3LC00630A-s001 [file LC-023-D3LC00630A-s001.pdf]

Electronic Supplementary Material (ESI) for Lab on a Chip.
This journal is © The Royal Society of Chemistry 2023

Driving electrochemical reactions at the microscale using CMOS microelectrode arrays

Supplementary Information

Jens Duru [a], Arielle Rüfenacht [a], Josephine Löhle [a], Marcello Pozzi [a], Csaba Forró [a], Linus Ledermann [a], Aeneas Bernardi [a], Michael Matter [a], André Renia [a], Benjamin Simona [b], Christina M. Tringides [a], Stéphane Bernhard [c], Stephan J. Ihle [a], Julian Hengsteler [a], Benedikt Maurer [a], Xinyu Zhang [a], and Nako Nakatsuka [a,*]

[a] Laboratory of Biosensors and Bioelectronics, Institute for Biomedical Engineering, Eidgenössische Technische Hochschule (ETH) Zürich, Switzerland
[b] Ectica Technologies AG, Zürich, Switzerland
[c] Macromolecular Engineering Laboratory, Department of Mechanical and Process Engineering, Eidgenössische Technische Hochschule (ETH) Zürich, Switzerland

[*] correspondence, E-mail: nakatsuka@biomed.ee.ethz.ch

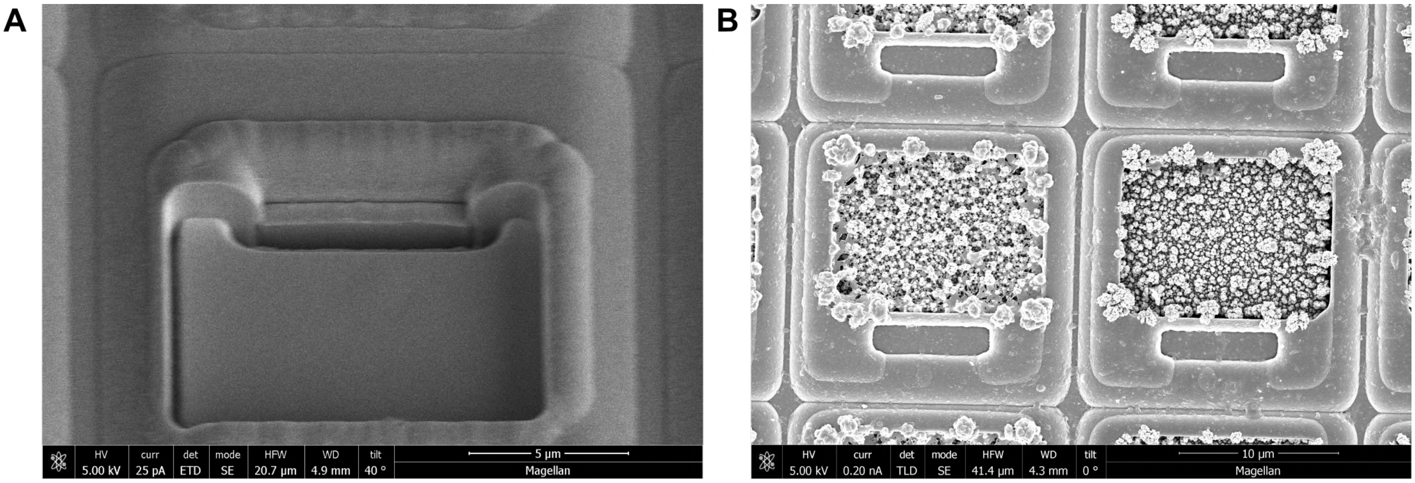

Figure S1: SEM images of a microelectrode on nonplanar CMOS MEAs with (A) bare platinum and with (B) platinum black-coated electrodes.

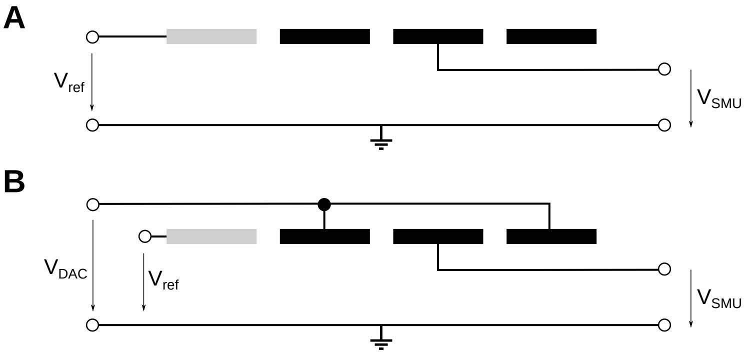

Figure S2: **Equivalent electrical circuits.** The circumferential reference electrode is shown in grey, while microelectrodes are shown in black. Between the reference electrode and the system ground, a voltage V_{ref} is measured, which in the standard case coincides with the mid-potential of the chip. An external voltage is applied via a source meter, providing the voltage V_{SMU}. The grounds of the MEA and the SMU are connected. **A** In case neighboring electrodes are left floating, the applied voltage between the cathode and the reference electrode (that serves as the anode as it is the second point in the electrochemical circuit with a defined potential) is defined by the difference of V_{SMU} and V_{ref}. Since the anode and cathode are not in close proximity, resulting regions of elevated pH are diffuse as shown in main text Fig. 3A/B. **B** In case neighboring electrodes are connected to a DAC with a defined voltage (V_{DAC}, in our case: mid-potential of the chip) the anode-cathode pair is in direct vicinity to each other and the potential difference is defined by the difference of V_{SMU} and V_{DAC}. This configuration leads to sharp regions of elevated pH as shown in main text Fig. 3C/D.

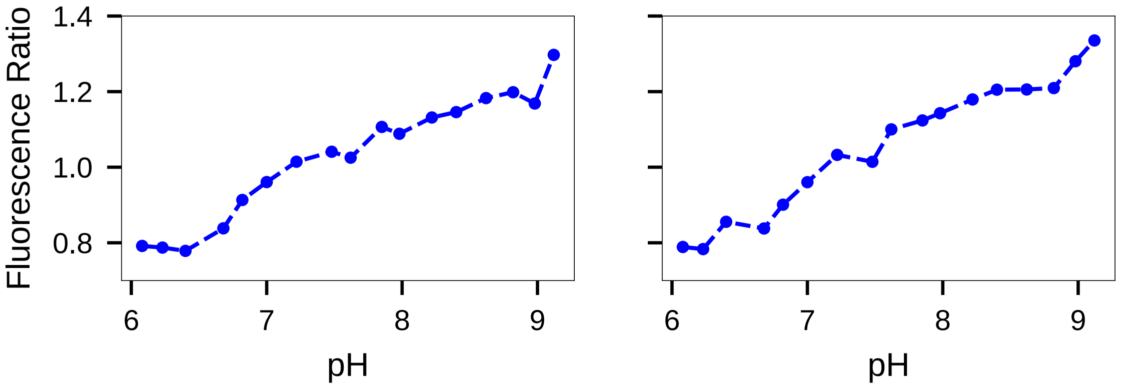

Figure S3: **Baselines for two PtB CMOS MEAs**. SNARF solutions (17) were manually adjusted to a pH within a range of 6-9 and incubated on the surface of CMOS MEAs. The relationship between pH and fluorescence ratio was obtained through dual-window imaging, which yielded a linear relationship.

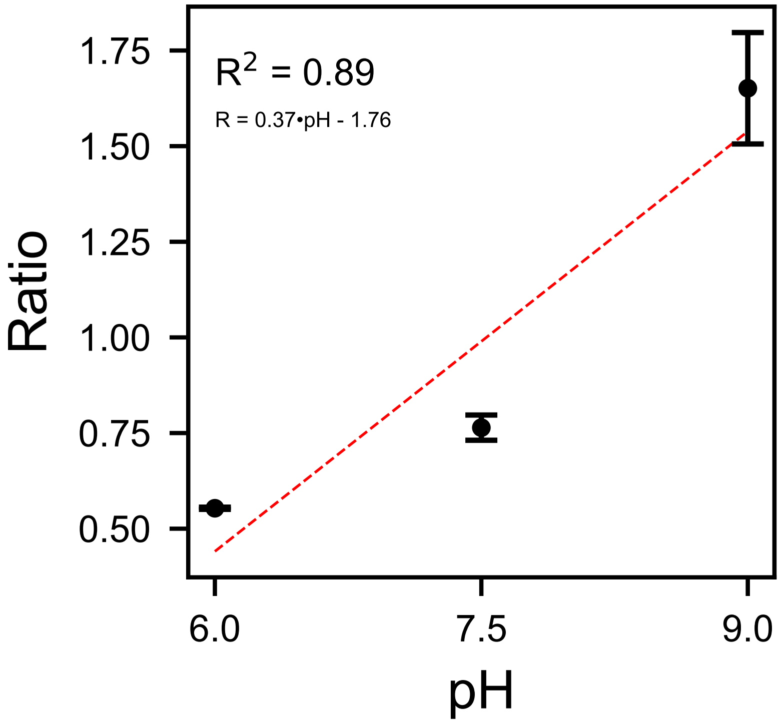

Figure S4: **Relationship between pH and fluorescent ratio and fit used for pH quantification.** Three images on different locations on two PtB CMOS MEAs were acquired and a linear fit was performed using linear regression on the resulting fluorescent ratios. The error bars indicate the standard deviation.

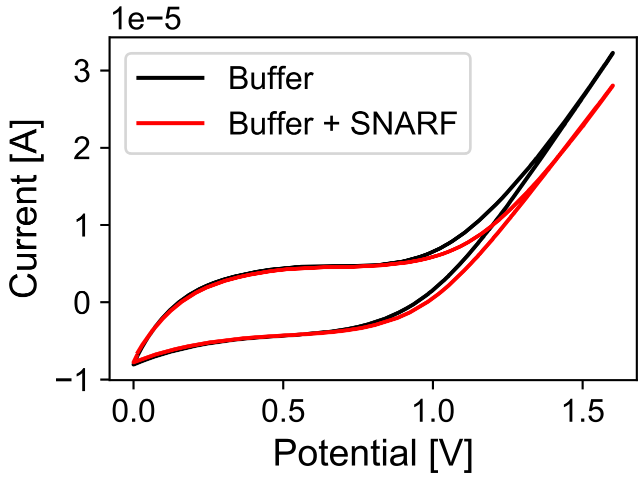

Figure S5: **Testing the electroactive properties of SNARF using cyclic voltammetry (CV).** The measurements were performed using an Autolab potentiostat (PGSTAT302N). A three-electrode system was set up with a platinum wire serving as the working electrode. Another platinum wire served as a counter electrode and an Ag/AgCl electrode as the reference electrode. All electrodes were placed in a well of a 12-well plate, which was filled with either the pure buffer solution or the buffer solution with SNARF added at concentrations mentioned in the "Materials and Methods" section of the main manuscript (55 μM). The potential was cycled three times between 0 and 1.6 V with a speed of 200 mV/s. Here, the last cycle is shown for both cases. No oxidation or reduction peaks appeared after the buffer solution was replaced with the buffer solution containing SNARF (retaining the positions of the 3 electrodes), demonstrating that SNARF is not electroactive under the conditions tested in the manuscript.

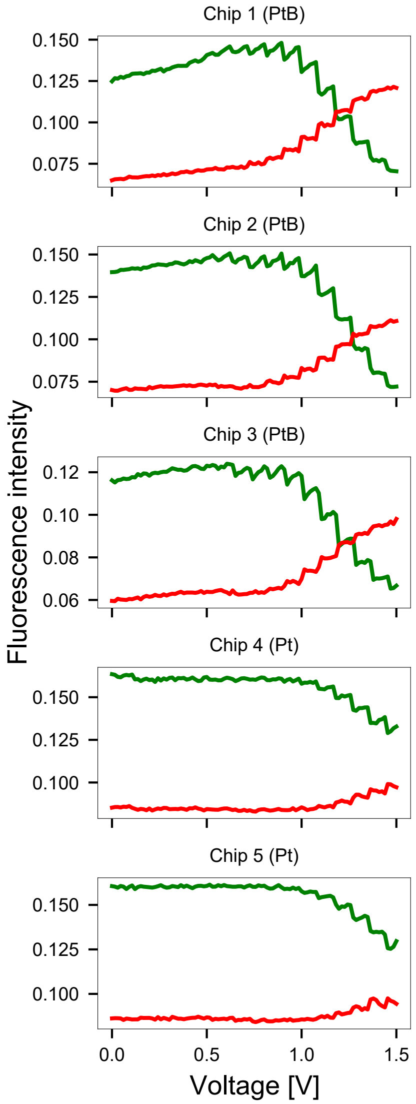

Figure S6: **Fluorescence emission of SNARF on three platinum black (PtB) and two platinum (Pt) chips.** The fluorescence intensity in the green window decreases with increasing pH. The intensity in the red window increases with increasing pH. PtB electrodes are able to induce larger pH changes.

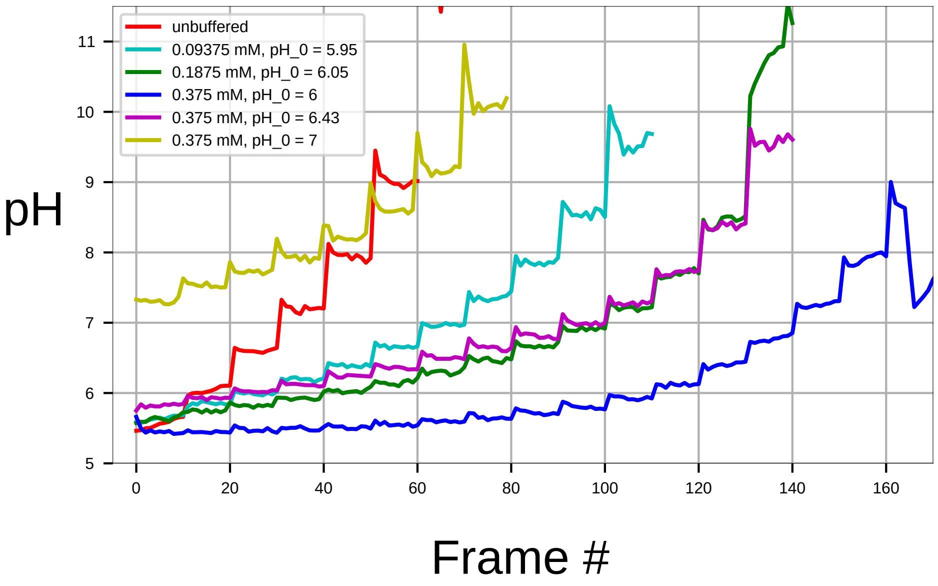

Figure S7: **Influence of buffer and starting pH on induced pH value**. Higher buffer concentrations require higher applied voltages to achieve a target pH. The voltage was changed from 0 (Frame 0) to 1.6 V (Frame 160) in steps of 0.1 V every 10 Frames (1 Frame = 15 s). Larger initial pH values ("pH_0") result in larger final pH values. These results were obtained using a non-linear relationship between fluorescence ratio and pH, hence values outside of the domain of the fit can be calculated.

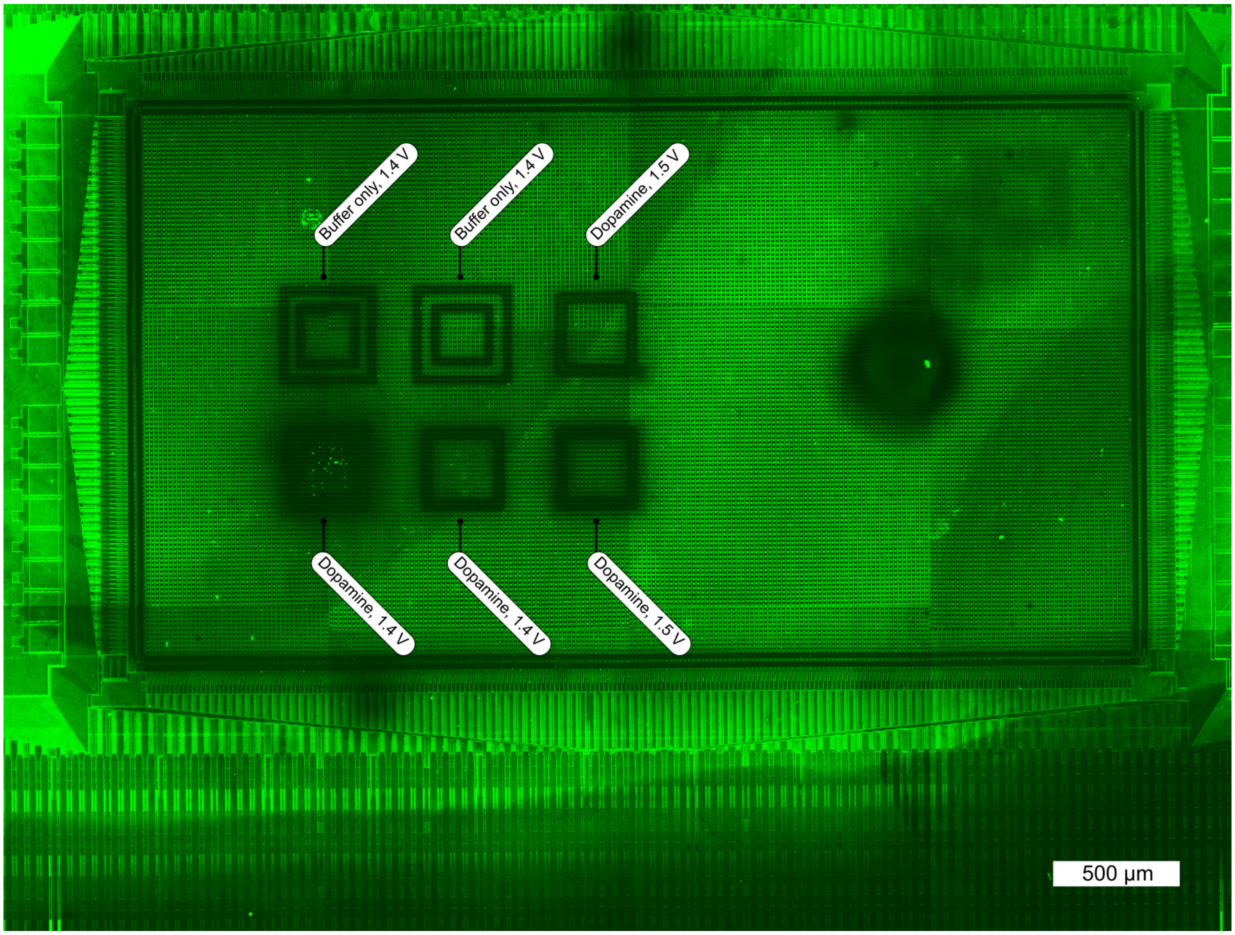

Figure S8: **Whole chip image after polydopamine deposition**. The labeled voltage was applied for 30 min at each square. When dopamine was present in the solution, dark squares were formed on the FITC-PLL-coated CMOS surface. When only the buffer was present, a hollow square appeared indicating the removal of PLL.

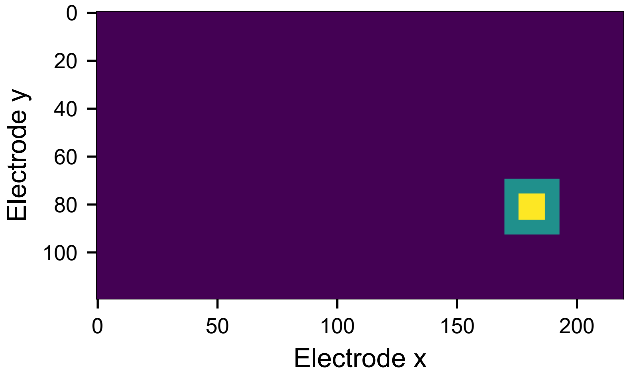

Figure S9: **Electrode routing for PLL removal experiments** The CMOS MEA consists of 26,400 electrodes that are arranged in a 120×220 grid. For the PLL delamination experiments shown in this work, the illustrated electrode configuration was used. The anode (shown in turquoise) was connected to a DAC, while the cathode (yellow) was connected to the SMU. The PLL was removed on the anode upon application of a positive voltage due to electrostatic repulsion.

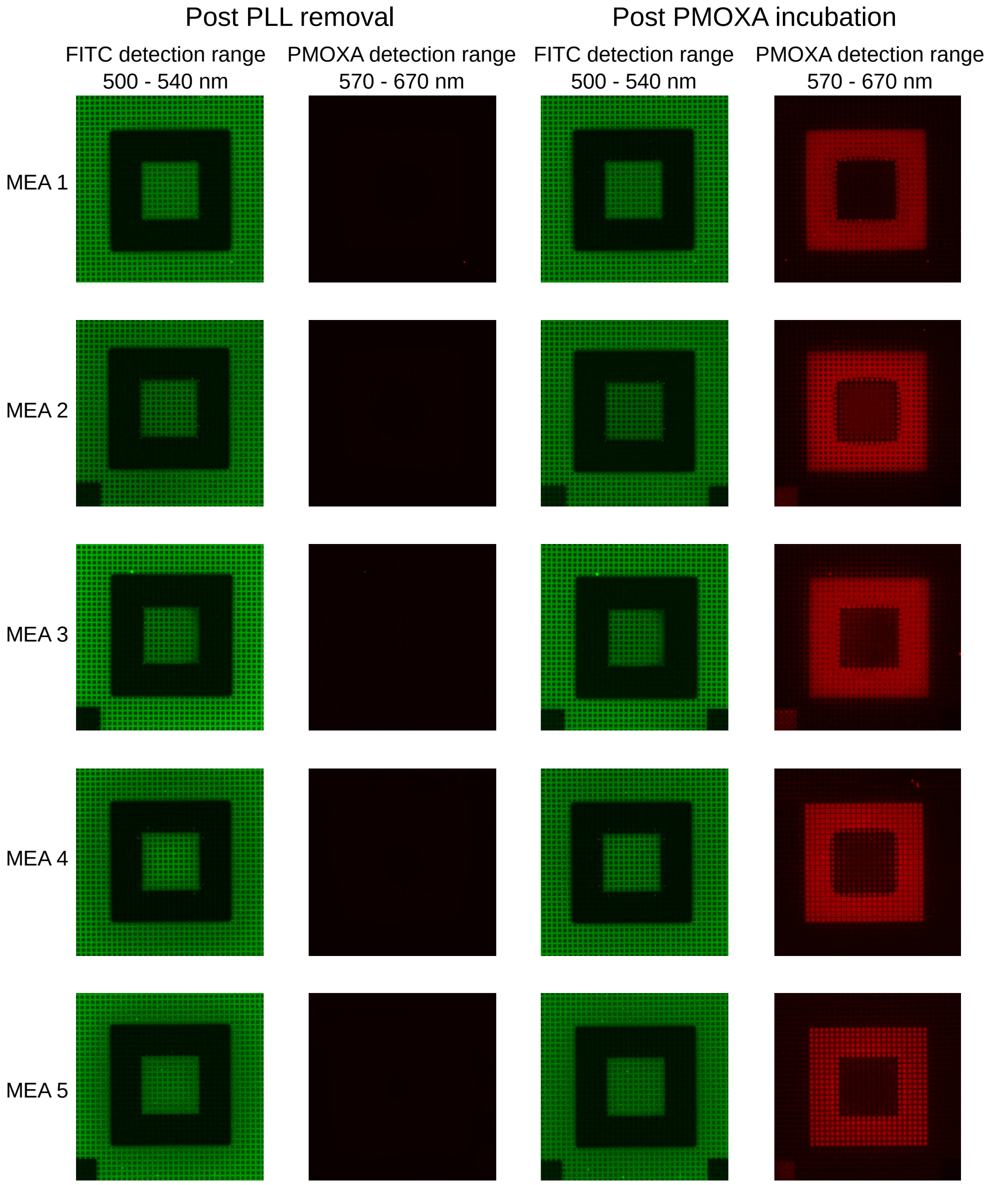

Figure S10: **Fluorescence emission post PLL removal and after PMOXA addition** FITC-PLL was removed from five CMOS MEAs (two left panels) and the voids were backfilled with PMOXA (two right panels). The small black squares in the left corner were induced through photobleaching.

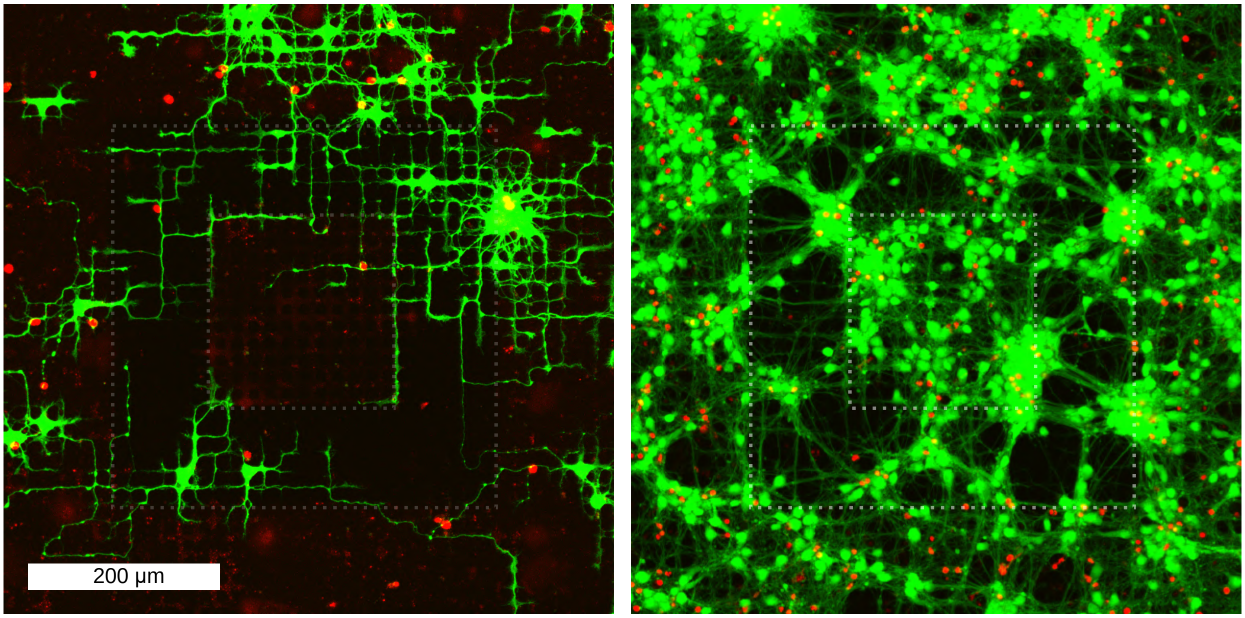

Figure S11: **Examples for cell overgrowth on the PMOXA patterns.** Axons are able to grow in between electrodes (left, 10,000 neurons were seeded) and at higher cell densities (right, 30,000 neurons were seeded=, neurons bridge the PMOXA pattern. The PMOXA outline is indicated by dashed white lines.

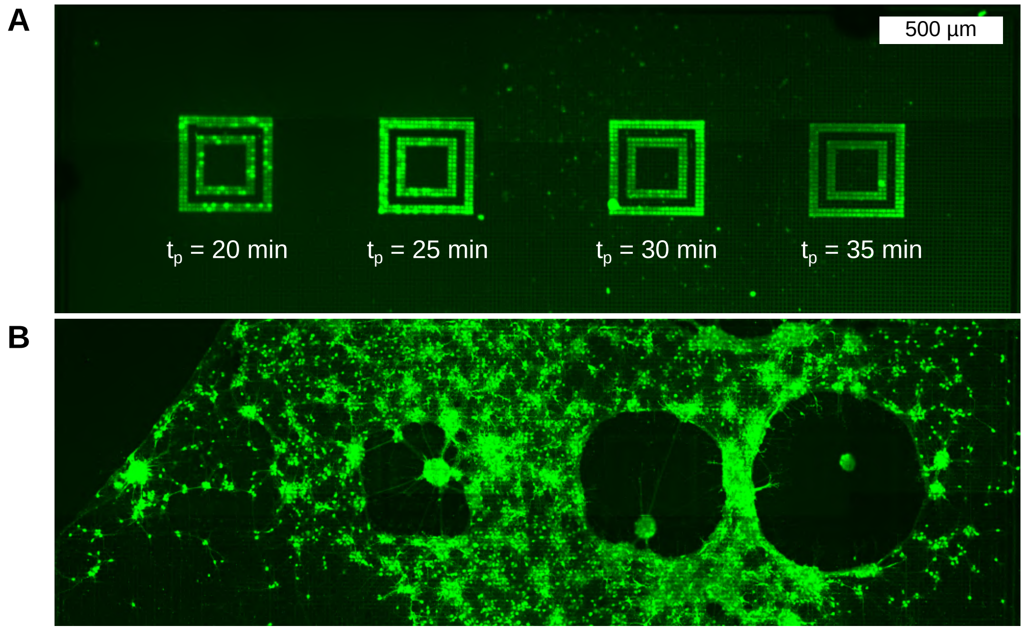

Figure S12: **Polymerization of hydrogels on the CMOS MEA. A** Enzymatically-crosslinked polyethylene glycol (PEG) hydrogel was polymerized on the CMOS MEA for different polymerization times ranging from 20 - 35 min. The hydrogel precursor solution consisted of 1.5% PEG dissolved in 0.125 mM HEPES/sodium acetate buffer. Lysine-tagged fluorescein isothiocyanate (Lys-FITC, 10 µM) was added to the precursor solution (1:12.5 v/v ratio) to render the hydrogel fluorescent upon its incorporation into the mesh. The resulting solution was then mixed in a 25:1 v/v ratio with fibrin stabilizing factor XIIIa and the pH was manually adjusted to 5.8. All chemicals were purchased from Ectica Technologies, Switzerland. Post polymerization, the FITC-lysine was found to be attached to the anode. **B** Primary rat cortical neurons at day in vitro 10 stained with CellTracker Green (CMFDA) dye avoid locations where hydrogels were grown. The extent of the avoidance-zone increases with hydrogel polymerization time.
